# Effectiveness of an integrated multilevel early child development intervention on caregiver knowledge and behavior: a quasi-experimental evaluation of the Malezi program in Tanzania

**DOI:** 10.1186/s12889-022-14956-2

**Published:** 2023-01-04

**Authors:** Gretchen Antelman, Josephine Ferla, Michelle M. Gill, Heather J. Hoffman, Teopista Komba, Amina Abubakar, Pieter Remes, Ola Jahanpour, Martha Mariki, Mary A. Mang’enya, Roland van de Ven

**Affiliations:** 1 Elizabeth Glaser Pediatric AIDS Foundation, Dar es Salaam, Tanzania; 2 Elizabeth Glaser Pediatric AIDS Foundation, Washington DC, USA; 3 The George Washington University, Milken Institute School of Public Health Washington DC, Washington DC, USA; 4 Institute for Human Development Aga Khan University (South-Central Asia, East Africa, UK), Nairobi, Kenya; 5 Development Media International, Mwanza, Tanzania; 6 President’s Office Regional and Local Government, Dodoma, Tanzania; 7 Ministry of Health, Dodoma, Tanzania

**Keywords:** Early child development, Parenting, Media intervention, Community health workers, Program evaluation, Caregiver

## Abstract

**Background:**

The quality of caregiving and the parent-child relationship is critical for early child development (ECD) and has been shown to be modifiable. This study evaluated an ECD project in Tanzania, assessing the effectiveness of *radio messaging* (RM) alone and a combined *radio messaging/video job aids/ECD* (RMV-ECD) intervention.

**Methods:**

This two-arm pre-post evaluation study enrolled a cohort of caregivers of children 0–24 months in four districts of Tabora region, following them for 9 months. ECD radio messages were broadcast on popular stations at least 10 times/day reaching all study districts. In two districts, community health workers (CHW), trained in UNICEF’s Care for Child Development package, used ECD videos in home- and facility-based sessions with caregivers. We used McNemar’s testing (pre-post pairs) within intervention group to describe how the intervention was associated with change in five outcomes: ECD knowledge, early stimulation, father engagement, responsive care, and environment safety. Logistic regression was used to describe the relative benefits of the combined intervention package (RMV-ECD) compared to radio messaging (RM).

**Results:**

In the RMV-ECD arm, all outcomes at endline except environment safety significantly improved after the intervention with the largest change seen in ECD knowledge (35.8% increase, *p* < .0001) and the smallest in father engagement (6.7%, *p* = .015). In the RM arm, ECD knowledge (5.7%, *p* = .031) and environment safety (18.1%, *p* = <.0001) improved. High measures of parenting stress were associated with lower likelihood of having good ECD knowledge (AOR 0.50, 95%CI: 0.35, 0.71), father engagement (AOR 0.72, 95%CI: 0.52, 0.99) and responsive care (AOR 0.31, 95%CI: 0.18, 0.54).

**Conclusions:**

An intervention that includes mass media, educational video content and CHWs who counsel caregivers in their homes and health facilities was associated with significant improvements in ECD parenting knowledge and behaviors but a relationship with responsive care could not be established. The less costly mass media-only intervention was associated with improved parenting knowledge and household environment safety. Parenting interventions targeting young children could be improved by incorporating more messaging and caregiver coaching in managing parental stress.

**Trial registration:**

NCT05244161 (17/02/2022); retrospectively registered with the US National Institutes of Health ClinicalTrials.gov.

## Introduction

A lack of nurturing care, malnutrition, and socioeconomic disadvantage [1] drives an acute need to advance child developmental outcomes in sub-Saharan Africa, a region reported to have more than three times the prevalence (66%) of at-risk children facing threats to their early cognitive and socio-emotional development [2, 3], compared to Europe/Central Asia (19%) and Latin America/Caribbean (18%). Responsive, sensitive care in the earliest years of life is critical for all domains of early child development (ECD), and for later school achievement and economic productivity in the adult years [2, 4, 5]. Further, the quality of caregiving and the parent-child relationship is modifiable [6–10]. Systematic reviews [11, 12] have shown that interventions aimed at supporting child development are successful in a variety of cultural and socio-economic contexts [7, 8], particularly in low to middle income countries [11].

There is a growing understanding, as documented in the 2018 Nurturing Care Framework, [13] that the integration of health, nutrition, responsive caregiving, safety and security, and early learning interventions into existing services is optimal for reaching children during the critical period from infancy to 3 years of age [7, 13, 14]. Interventions limited to health facility settings have demonstrated that audio-visual job aids can be effective education tools across a wide range of health topics [15] and a cost-effective way to reach caregivers [16]. But the use of multiple entry points, such as leveraging opportunities to engage community members through outreach or mass media, is critical to broadening the reach and impact of child development interventions [4, 5, 7, 8, 12, 17]. Several studies have demonstrated that integrated, community and facility-based interventions can improve child nutrition and growth [18]; parenting behaviors [16, 19]; and cognitive and motor development [20–24]. In order to reach a broader audience, including family/household influencers such as fathers or grandparents, mass media radio messaging campaigns can increase caregiver and community knowledge, and caregiver motivation to seek appropriate health care [25–27]. A recent review emphasized the need for newborn policies in Asian, Africa and Latin America to recognize and harness the role of the grandmother in affecting child health and establishing parenting norms and behaviors [28].

Despite strong economic growth over the past decade, one-quarter of Tanzanians live in poverty [29], and about 70% of children in Tanzania are at risk of poor development [1]. Radio is the most frequently accessed form of media among both women and men; 45% of women and 60% of men listen to the radio at least once a week [30]. The Government of Tanzania has shown a commitment to promoting ECD policies [31, 32], and community health workers (CHW) have recently become a national cadre within the government’s health system. While this new cadre and implementation of child development policy frameworks have not yet matured to scale, evaluations of programs that leverage broadening access to mass media and capacitate CHWs to support integrated child development interventions in Tanzania are especially timely.

The Malezi (*caring for young children* in Swahili) Project in Tabora, Tanzania started in 2016 to integrate the promotion of ECD practices in facility and community settings. In phase I, the project supported the integration of an ECD package -- based on UNICEF’s Care for Child Development (CCD) package, adapted for Tanzania -- into pregnancy and under-5 health services in selected districts of Tabora region. The training was focused on building providers’ counseling skills and understanding the concept of responsive caregiving. The project also provided paper job aids, toys, and logistical/technical support for ongoing mentorship structures. After demonstrating that the Malezi I intervention was feasible and acceptable, the Malezi II Project was designed to expand geographic coverage of Malezi I in 2019 and augment the intervention components by introducing video job aids and mass media ECD messaging; and build in a more rigorous program evaluation. This Malezi II evaluation study was designed to compare outcomes associated with two interventions, radio messaging alone and a combined ECD package/video job aids/radio messaging intervention. Study outcomes were ECD knowledge, early stimulation, father engagement, responsive caregiving, and household environment safety.

The theory of change underlying the combined intervention design drew from current evidence for parenting interventions [7, 12] and behavior change literature [33]. It proposed that a combination of caregiver attitudes and norms, informed by access to information at individual and community level, would increase caregiver knowledge, influence caregiver intentions, and ultimately their behaviors. While caregivers may develop an intention to change through program support, actual behavior change could be limited by a lack of skills or a less-enabling environment. We hypothesized that the combined intervention would have a stronger impact on caregiving behaviors, compared to radio messaging alone. Since the comprehensive Malezi II program model is resource intense, our study was designed to describe the relative benefits of the comprehensive intervention to change caregiver behaviors, compared to the benefits of radio messaging alone, which is potentially more efficient and scalable.

## Methods

### Design and setting

We conducted a two-arm quasi-experimental pre-post evaluation study, comparing different 9-month intervention packages to establish their relative impact on parenting skills and environment among a cohort of caregivers of children under age 3 years in Tabora region, located in central-western Tanzania and home to a predominantly rural (87%) population and agricultural economy [34]. In Tanzania, about 8 in 10 adults are literate (77% of women and 83% of men), with about half having completed primary education, and 23% of women and 28% men having secondary or higher education [30]. The study included four districts purposefully divided into two intervention groups. The first group (Kaliua, Uyui districts) was exposed to the *minimal intervention package,* composed of radio messaging (**RM**) only. The second group (Nzega, Igunga districts) was exposed to the Malezi II *full intervention package,* composed of radio messaging, the introduction of short video job aids primarily for CHW use, and the CCD program (**RMV-ECD**), first implemented under the Malezi I phase and continued under Malezi II.

### Intervention

#### Radio messaging (RM)

ECD radio messages (37 different spots) were aligned with Government policy and community tested prior to being aired on the three most popular radio stations in Tabora at least 10 times per day from March to December 2020. These spots focused on the importance of playing, talking with, and praising young children, using positive discipline, and the importance of both mothers and fathers interacting with young children. Radio messaging was targeted to reach all four study districts as much as possible, though coverage of some stations varied by district and proximity to urban centers.

#### CCD program (RMV-ECD)

In addition to radio messaging, caregivers in this arm were also allocated to a CHW for monthly household visits. These CHWs received training on CCD and on how to use five short (5–6 minutes) ECD video job aids loaded onto electronic tablets in individual home sessions. The short videos were also used during group counseling sessions at clinics but this was not limited to the RMV-ECD arm caregivers. Videos were produced in Tabora and showed local caregivers and community health workers demonstrating recommended ECD practices (Swahili videos with English subtitles can be viewed at https://www.developmentmedia.net/project/malezi-ii/). Four of the videos concentrated on nurturing care practices specific to age groups (0–6 months, 6–12 months, 12–24 months, 24–36 months) and one video covered cross-cutting issues, applicable to all ages. Intervention fidelity monitoring data were collected monthly to document completion of CHW home visits to assigned caregivers.

All ECD radio messaging and video (job aid) content was developed through an iterative process informed by target community members including mothers, fathers, and elders who are influential stakeholders with regard to perpetuating community and family norms around child-rearing. Content development was led by the Development Media International (DMI) who deployed behavioral media specialists to conduct focus group discussions (FGD) aiming to understand how the content is being received by the target audience of caregivers; and investigate whether individuals who have heard the ECD content can identify the norms and behaviors that it is aiming to address.

### Sample and sampling procedures

As the comprehensive program intervention was primarily delivered by CHW who were affiliated with health facilities, we purposefully selected 31 health facilities located in 29 administrative units called wards (6–8/district). From these wards, 75 National Bureau of Statistics census enumeration areas (EA) were randomly sampled proportional to population size. In order to minimize selection bias in participant recruitment, the study team aimed to enumerate all households in sampled EAs, listing potentially eligible households if there was a resident adult (> 18 years) who was a primary caregiver of a child aged 0–24 months and who intended to remain in the same area for at least 1 year, and was willing to be home-visited by a CHW. From these listed households, only one primary caregiver per household was recruited by a study enumerator. Caregivers who were not able to provide written informed consent due to a cognitive impairment or language barrier; or who were the primary caregiver of an index child with a congenital anomaly or other disability; or who worked as a CHW or medical provider, were excluded from the study.

**Fig. 1:**
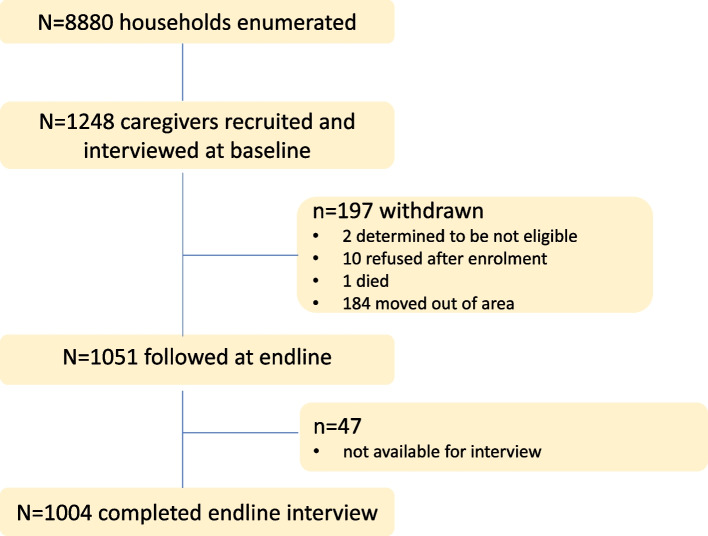
Study Consort

We estimated that a sample size of 430 caregivers per intervention group would provide 90% power to detect a 15% difference between the RM and RMV-ECD intervention groups at endline, and > 80% power to detect at least a 5% change in each intervention group between baseline and endline, at a 5% significance level. Of 8880 households enumerated, 1248 caregivers were recruited into the study and interviewed at baseline (October–December 2019). Among these, 12 were withdrawn (10 refused after enrolment; two were excluded after being found ineligible for the study); one caregiver died; and 184 caregivers moved out of the area. Of the remaining (*n* = 1051) eligible for follow-up, 47 (4%) were not available for interview after several attempts, and 1004 (96%) were successfully interviewed at endline (January–March 2021; Fig. 1), which was 17% higher than our minimum required sample size. Almost all (*n* = 985; 98%) caregivers interviewed at endline remained the primary caregiver of the index child from baseline. Of the 19 caregivers whose index child had died or moved from the household, eight nominated an eligible “replacement” child under 3 years and 11 completed a partial interview skipping questions that were no longer applicable.

### Data collection and study variables

Baseline and endline structured questionnaires were administered via interview in a private place in or near the consenting caregiver’s home in the national language (Swahili). Interviews assessed exposure to the intervention and caregiver knowledge/practices using questions tailored to the study intervention. Responses were based on caregiver self-report and interviewer observations and categorized according to five outcome variables reflecting caregiver knowledge, stimulation practices, father engagement, responsive care, and household environment safety. Continuous scores for each variable were dichotomized at the median for analysis. Some scores, where the number of items differed by age of the child (early stimulation) or sub-group (environment risk), were standardized to a 0–1 scale by dividing the raw score by the number of items.

Age-appropriate ECD knowledge was assessed from six questions (scoring 0–6 points) asking the caregiver to describe one specific way that a “caregiver can support a child’s mental, emotional or physical development …” during pregnancy, from birth to 6 months, from 6 to 9, 9–12, and 12–24 months and 2–5 years of age. Caregiver responses were recorded by the interviewer verbatim and coded by the co-Principal Investigator (JF) as correct or incorrect. Non-specific responses such as “seeking health care” were not considered correct.

Early stimulation practice and father engagement measures were adapted from questions originating from UNICEF’s Multiple Indicator Cluster Surveys (MICS), a widely validated survey used in over 100 countries over the past two decades [35–37]. Caregivers of children under 7 months could score up to three points for reporting that the mother, father, or other adult engaged the child in singing songs, taking the child outside, or playing with the child in the past week. Caregivers of children over 7 months were asked three additional items (read books, told stories, name/count things with child) for a total of six points. The sum of the items for these two measures were then standardized to a 0–1 scale.

Responsive care was defined for caregivers of children over 7 months based on interviewer observations of how the caregiver engaged with the child during the interview. This measure had a high proportion of missing data (21% at baseline, 24% at endline) due to children being too young, sleeping or absent during the interview. The four items totaling up to six points included helping the child keep busy (0, 1), pointing out objects/naming things (scored as 0, 1), recognizing when the child needs help with something (0,1,2), and keeping the child in view at all times (0,1,2).

Household and neighborhood environment safety risks were assessed by interviewer observation of the inner and outer household areas, where risks were grouped by community (nearby road, bar/market, ditches); outside compound (open water source, unpenned animals, accessible sharp tools, chemicals or flammable materials, and unprotected cooking area); and inside household (accessible electric, medicine or cleaning chemicals, inappropriate toys). The environment safety outcome score was standardized (0–1) to adjust for the different number of items in each group.

Several variables were explored to describe potential and actual exposure to the intervention. Radio ownership and recent CHW visits were assessed at baseline and endline in both study groups. Radio message content recall and frequency of radio listenership were assessed at endline in both study groups. Exposure to the intervention videos through home and facility visits was assessed in the RMV-ECD arm only at endline, and overall number of CHW visits was assessed in both study groups.

Other predictor and mediator variables including social-demographic characteristics included history of child illness/injury, health care utilization, parental discipline practices/beliefs, parenting stress, and depressive and anxiety symptoms. Health care utilization and parental discipline measures were adapted from the UNICEF MICS tool, where the discipline assessment contained 11 items, each answered “yes” or “no,” and divided into four sub-groups: psychological, physical, severe physical and positive (non-violent) disciplinary practices by anyone in the household in the past month. The eight violent discipline items comprise the measure on violent discipline, and the three remaining non-violent items comprise the “positive discipline” measure. Discipline scores were standardized to a scale of 0–1.

The Parenting Stress Index (PSI) scale is a widely used and robust measure of three parenting related domains: Parental distress, parent-child relationship dysfunction (e.g. quality of relationship), and the extent to which the caregiver perceives the child is difficult [38]. The scale is composed of 36 statements (12 per sub-scale domain) which are scored 1 (strongly disagree) to 5 (strongly agree) and can be summed to reflect the total score for each domain. The PSI-36 total score is a composite score of the three subscales (scores range 36–180) with higher scores (or a cut-off of 90) indicating higher parental stress.

Depressive symptoms were measured using the 9-item Patient Health Questionnaire, a tool widely used in resource-limited settings and recently validated in a Tanzanian primary care population, showing 78% sensitivity and 87% specificity in detecting depression when compared to a gold-standard psychiatric assessment [39]. The General Anxiety Scale, often used in low to middle income country settings [40, 41], measures the seven criteria of anxiety in the Diagnostic and Statistical Manual of Mental Disorders, establishing a provisional anxiety diagnosis and assessing symptom severity [42].

### Statistical analysis

We summarized caregiver characteristics at baseline, and measures of covariates and intervention exposure at baseline and endline using frequencies and percentages for categorical variables and means, medians, interquartile ranges (IQR), and standard deviations (SD) for continuous variables. Participant characteristics and baseline outcome measures in the two intervention arms were compared using Chi-Square or Rank Sum tests. We limited all analyses to those who were followed at endline, after conducting an attrition analysis which showed minimal statistically significant differences between those excluded and included (data not shown).

Outcomes associated with the intervention were assessed using McNemar’s test and further described by the proportion of caregivers who improved from having a poor outcome score at baseline to having a good score at endline, with 95% confidence intervals (CI) to allow comparison of interventions by study arm.

Unadjusted and adjusted logistic regression models that accounted for sampling weights and clustering by sampling unit were used to describe the association between the full intervention arm, (RMV-ECD) compared with the the radio-only (RM) arm on study outcomes, as well as other covariables of interest. Baseline status of outcomes of interest and child age were included in adjusted models. All data management and descriptive analyses were done using Stata 16.1; McNemar’s and regression analyses were done in SAS 9.4.

### Ethical considerations

The protocol for this evaluation was approved by the National Research Ethics Committee of the Tanzania National Institute of Medical Research and the Advarra Review Board in the United States. Study interviewers obtained written informed consent from caregivers enrolled in the study prior to conducting the baseline interview.

## Results

### Caregiver and index child characteristics

The majority of enrolled caregivers (*n* = 1004) were biological mothers (97.9%) with a median age of 26 years and most (84.5%) were married or cohabiting (Table 1). Almost one in five (19.2%) had not completed primary education; fewer (10.9%) had more than a primary level education. Only a few characteristics differed between the study arms. Caregivers in the RMV-ECD arm were more often partnered with more educated partners (22.5% vs 15.8%), had fewer under-5 children in the household (3+ children: 7.9% vs 16.5%), and were less likely to be farmers 61.7% vs 74.1%), compared to caregivers in the RM arm.

**Table 1:** Caregiver characteristics at baseline, overall and by study arm

		Overall (***n*** = 1004)	RM only (***n*** = 484)	RMV-ECD (***n*** = 520)	***p***-value
		*N* (%) or median (IQR)			
Caregiver age in years	Median (IQR)	26 (22, 31)	26 (22, 31)	27 (22, 32)	0.125
Caregiver sex	Female	1001 (99.7)	484 (100.0)	517 (99.4)	0.094
Relation to index child	Mother	983 (97.9)	479 (99.0)	504 (96.9)	0.052
	Grandmother	18 (1.8)	5 (1.0)	13 (2.5)	
	Father	3 (0.3)	0 (0.0)	3 (0.6)	
Education	Not completed primary	193 (19.2)	97 (21.1)	96 (18.5)	0.146
	Primary	700 (68.9)	343 (71.0)	357 (68.8)	
	Secondary / higher	109 (10.9)	43 (8.9)	66 (12.7)	
Has partner (married, cohabiting)		848 (84.5)	417 (86.2)	431 (82.9)	0.153
Partner education	None	110 (13.0)	56 (13.4)	54 (12.5)	0.048
	Primary	575 (67.8)	295 (70.7)	280 (65.0)	
	Secondary/higher	163 (19.2)	66 (15.8)	97 (22.5)	
	No partner	156 (−-)	67 (−-)	89 (−-)	
Household living situation	Nuclear family	636 (63.3)	301 (62.2)	335 (64.4)	0.463
	Extended family	368 (37.7)	183 (37.8)	185 (35.6)	
Number of children under 5 in household	1	478 (47.6)	201 (41.5)	277 (53.3)	<.0001
	2	405 (40.3)	203 (41.9)	202 (38.9)	
	3+	121 (12.1)	80 (16.5)	41 (7.9)	
Household income source	Farming	678 (67.7)	358 (74.1)	320 (61.7)	<.0001
	Self-employ/informal (business, laborer)	254 (25.3)	99 (20.5)	155 (29.9)	
	Formal (professional)	38 (3.8)	14 (2.9)	24 (4.6)	
	No work	32 (3.2)	12 (2.5)	20 (3.9)	
	Missing	2 (−-)	1 (−-)	1 (−-)	
Number of assets (bicycle, motorcycle, car/truck, animal plough, tractor/tiller, television, computer/tablet)	None	235 (23.4)	120 (24.8)	115 (22.1)	0.073
	1–2	692 (68.9)	319 (65.9)	373 (71.7)	
	3–7	77 (7.7)	45 (9.3)	32 (6.2)	

Index children of caregivers were a median of 11–12 months of age at recruitment and 26 months at follow-up (Table 2). Injury requiring medical attention in the past year was not uncommon ranging from 12.5% in RMV-ECD arm at baseline to 24.0% of the RM arm at endline. The median parenting stress index score ranged from 70 to 72 across arms and time periods, and the proportion scoring above the cut-off (> 90) for high parenting stress was 19.0% (RM) and 20.2% (RMV-ECD) at baseline. This decreased slightly in both arms to 15.7 and 11.7% at endline. Most caregivers had no depressive or anxiety symptoms. Discipline scores, standardized to a scale of 0–1, may be interpreted as the proportion of caregivers who reported “yes” to one or more items within the subscale. Non-violent discipline practices were the most common and severe physical discipline was the least common type of discipline practiced. Violent and non-violent discipline subscale scores increased over time and in both arms, although this increase was confounded by child’s age (data not shown). At endline, the RM arm had statistically higher rates of psychological (0.48) and physical discipline (0.43) practices compared to the RMV-ECD arm (0.37 and 0.34 respectively; *p* < .0001).

**Table 2:** Caregiver characteristics at baseline and endline, by study arm

		Baseline (***n*** = 1004)			Endline (***n*** = 1004)		
		RM only (***n*** = 484)	RMV-ECD (***n*** = 520)	***p***-value	RM only (***n*** = 484)	RMV-ECD (***n*** = 520)	***p***-value
		*N* (%) or other, specified			*N* (%) or other, specified		
Caregiver’s index child age in months	Median (IQR)	12 (6, 16)	11 (6, 19)	0.299	26 (20, 31)	26 (21, 34)	0.132
Any under-5 years child in household had injury requiring medical care in past year	No	413 (85.3)	455 (87.5)	0.316	368 (76.0)	418 (80.7)	0.073
	Yes	71 (14.7)	65 (12.5)		116 (24.0)	100 (19.3)	
Child has had expected number of under-5 clinic visits since birth	No	347 (71.7)	338 (65.0)	0.023	294 (60.7)	251 (48.3)	<.0001
	Yes	137 (28.3)	182 (35.0)		190 (39.3)	269 (51.7)	
Parenting Stress Index (PSI) (36 items, possible range 36–180)	Median (IQR)	72 (59, 86)	70 (57, 86)	0.486	72 (62, 83)	71 (59, 82)	0.135
PSI sub-scales (12 items each; possible range 12–60)	Parental distress	28 (22, 36)	28 (21, 36)	0.837	26 (21, 32)	26 (21, 31)	0.468
	Dysfunction	19 (14, 23)	19 (14, 24)	0.770	21 (16, 24)	20 (16, 23)	0.200
	Difficult Child	24 (19, 29)	23 (18, 29)	0.064	25 (21, 30)	25 (20, 29)	0.060
PSI cutoff: > 90^a^	No	392 (81.0)	415 (79.8)	0.637	396 (84.3)	432 (88.3)	0.065
	Yes	92 (19.0)	105 (20.2)		74 (15.7)	57 (11.7)	
PHQ Depressive symptoms	None (< 5)	355 (69.2)	335 (64.4)	0.108	312 (64.5)	306 (58.9)	0.184
	Mild (5–9)	109 (22.5)	149 (28.7)		139 (28.7)	172 (33.1)	
	Moderate (10–14)	29 (6.0)	29 (5.6)		22 (4.5)	25 (6.7)	
	Moderate/severe (15–19)	6 (1.2)	6 (1.1)		7 (1.5)	4 (0.8)	
	Severe (20+)	5 (1.0)	1 (0.2)		4 (0.8)	3 (0.6)	
GAD-7 Anxiety symptoms	None (< 5)	383 (79.1)	403 (77.5)	0.771	379 (78.3)	363 (69.8)	0.002
	Mild (5–9)	84 (17.4)	100 (19.2)		84 (17.3)	141 (27.1)	
	Moderate (10–14)	10 (2.1)	12 (2.3)		17 (3.5)	12 (2.3)	
	Severe (15+)	7 (1.4)	5 (1.0)		4 (0.8)	4 (0.8)	
Discipline subscales^b^ (mean, SD)	Psychological (2 items)	0.31 (0.38)	0.31 (0.35)	0.687	0.48 (0.36)	0.37 (0.34)	<.0001
	Physical (4 items)	0.24 (0.27)	0.24 (0.29)	0.743	0.43 (0.31)	0.34 (0.32)	<.0001
	Severe physical (2 (items)	0.02 (0.11)	0.03 (0.12)	0.509	0.07 (0.21)	0.08 (0.22)	0.941
	Non-violent discipline (3 items)	0.48 (0.38)	0.46 (0.39)	0.372	0.77 (0.28)	0.71 (0.30)	0.002

^a^At endline, *n* = 14 and *n* = 31 missing from RM only and RMV-ECD groups, respectively

^b^All discipline item (*n* = 11) sub-scales are standardized to 0–1 scale. Violent (negative) discipline behaviors (*n* = 2) are described by the psychological (shouted/yelled; called names), physical (*n* = 4; shook, spanked, hit on bottom, hit arms/legs) and severe physical (*n* = 2; beat up, hit in head/face) subscales, and together make the study outcome discipline variable. The non-violent discipline subscale includes positive discipline behaviors (*n* = 3; gave something else; explained why wrong; took away privileges)

### Intervention exposure

Proxy and direct measures of intervention exposure are described in Table 3. Radio access was lower in the RM arm (44.2%) compared to the RMV-ECD arm (56.5%, p < .0001) at baseline and while access improved in both arms, the differential between the two arms became more pronounced at endline. Similarly, at endline, 54.7% of the RM arm reported hearing or recalling the ECD radio message compared to 74.6% of the RMV-ECD arm (p < .0001), though slightly more caregivers (66.6%) in the RM arm reported hearing the messages at least weekly, compared to the RMV-ECD arm (58.6%, *p* = .056).

**Table 3:** Measures describing potential and reported intervention exposure by study arm

		Baseline (***n*** = 1004)			Endline (***n*** = 1004)		
		RM only (***n*** = 484)	RMV-ECD (***n*** = 520)	***p***-value	RM only (***n*** = 484)	RMV-ECD (***n*** = 520)	***p***-value
		*N* (%)			*N* (%)		
Radio access	None	279 (55.8)	226 (43.5)	<.0001	238 (49.2)	139 (26.7)	<.0001
	Outside household	19 (3.9)	46 (8.8)		44 (9.1)	86 (16.5)	
	Within household	195 (40.3)	248 (47.7)		202 (41.7)	295 (56.7)	
Malezi intervention radio message recall^a^	Not heard				219 (45.3)	132 (25.4)	<.0001
	Heard but cannot recall content				23 (4.7)	27 (5.2)	
	Heard and recalled content				242 (50.0)	361 (69.4)	
Frequency heard radio message	Daily				81 (36.7)	88 (26.0)	0.056
	Weekly				66 (29.9)	110 (32.6)	
	Monthly				45 (20.3)	88 (26.0)	
	< Monthly				29 (13.1)	52 (15.4)	
	Unknown				44 (−-)	50 (−-)	
Last CHW visit	Never	557 (94.4)	460 (88.5)	0.002	470 (97.1)	28 (5.4)	<.0001
	3+ months ago	10 (2.1)	11 (2.1)		9 (1.9)	97 (18.7)	
	1–3 months ago	11 (2.3)	33 (6.3)		4 (0.8)	208 (40.0)	
	< 1 month ago	6 (1.2)	16 (3.1)		1 (0.2)	187 (36.0)	
Exposure to video ever, in home or facility	No					66 (12.7)	
	Yes					454 (87.3)	
Exposure to videos in the home	Never					79 (15.2)	
	3+ months ago					135 (25.9)	
	1–3 months ago					146(28.1)	
	< 1 month ago					160 (30.8)	
Exposure to videos in the facility	Never					302 (58.1)	
	3+ months ago					78 (15.0)	
	1–3 months ago					58 (11.1)	
	< 1 month ago					82 (15.8)	

^a^For subsequent analyses, exposure to radio messaging reclassified as not heard (0) vs. heard (1 = recall or not) due to small numbers in the heard/no recall category

At baseline, where the RMV-ECD arm had benefited from the Malezi I program, there was a slightly higher rate of receiving a CHW visit in the past year (11.5%) compared to the radio-only arm (5.6%, *p* = .002). During the intervention in the RMV-ECD arm, each CHW was assigned a median of five (IQR 4,7) caregivers to visit monthly during the intervention. From March to November 2020, a total of 4536 CHWs visits to assigned caregivers were completed during the intervention period. Enrolled caregivers received a median of 8 (IQR 7, 9) visits during the intervention period and 84% received six or more visits. At endline, in the RM arm where CHW home visits were not supported, 2.9% reported a CHW home visit compared to 94.6% of the caregivers in RMV-ECD arm (*p* < .0001).

Remaining measures demonstrate the relatively high rate of exposure to the ECD videos (87.3%) in the RMV-ECD arm, though videos were more likely to have been seen recently at home visits rather than at facility visits.

### Intervention effects

We describe study outcomes in Table 4, first comparing RM and RMV-ECD arms at baseline (columns 2–4) to show the likely effect of the Malezi I intervention occurring prior to this study. ECD knowledge, early stimulation practices and “good” scores on environment/household safety are at significantly higher levels in the RMV-ECD arm compared to the RM arm at baseline; father engagement showed no difference and responsive care was lower in the RMV-ECD arm compared to the radio-only arm at baseline.

**Table 4:** Study outcomes at baseline, endline, and percent change by intervention arm

	Baseline (BL), ***n*** = 1004			Endline (EL), ***n*** = 1004					
**Column**	**2**	**3**	**4**	**5**	**6**	**7**	**8**	**9**	**10**
	**RM only**	**RMV-ECD**	*p*-value* (col 2 vs 3)	**RM only**		*p*-value** (col 2 vs 5)	**RMV-ECD**		*p*-value** (col 3 vs 8)
	***N*** **(%)**	***N*** **(%)**		***N*** **(%)**	**% increase from BL**		***N*** **(%)**	**% increase from BL**	
**Early child development knowledge**									
Poor (< 2)	350 (72.3)	332 (63.9)	0.004	322 (66.4)	+ 5.9	0.031	146 (28.1)	+ 35.8	<.0001
Good (2–6)	134 (27.7)	188 (36.1)		163 (33.6)			374 (71.9)		
**Early stimulation practices**									
Poor (< 50%)	317 (65.4)	289 (55.6)	0.001	315 (65.1)	+ 0.4	0.886	241 (46.5)	+ 9.1	0.042
Good (> 50%)	167 (34.5)	231 (44.4)		169 (34.9)			277 (53.5)		
**Father engagement**									
Poor (< 2)	319 (65.9)	339 (65.2)	0.811	344 (70.9)	−5.0	0.069	304 (58.5)	+ 6.7	0.015
Good (2–6)	165 (34.1)	181 (34.8)		141 (29.1)			216 (41.5)		
**Responsive care**									
Poor (< 5)	182 (50.1)	268 (62.2)	0.001	178 (50.4)	−.0.3	0.866	193 (46.5)	+ 15.7	0.0002
Good (5–6)	181 (49.9)	163 (37.8)		175 (49.6)			222 (53.5)		
**Environment/household safety**									
Poor (> 0.3)	295 (61.0)	201 (38.7)	<.0001	208 (42.9)	+ 18.1	<.0001	182 (35.0)	+ 3.7	0.213
Good (< 0.3)	189 (39.0)	319 (61.3)		277 (57.1)			338 (65.0)		

* Chi-square test of distributions of caregivers in MR and RMV-ECD arms at baseline (independent samples)

** McNemar’s test of caregiver pairs (dependent samples)

Description of study outcomes (cut-point for categorical variables is median and each item scores 1 point, unless otherwise specified):

• ECD knowledge: Number of known age-appropriate stimulation activities (6 items)

• ES practices: Proportion of age-appropriate stimulation activities done with child on a weekly basis (3 items for infants < 6 months; 6 items for 6+ months; standardized score to 0–1 scale)

• Father engagement: Number of activities father engaged in past week (6 items; cut-point at top tertile)

• Responsive care: Interviewer-observed responsive care behaviors (4 items, scoring 0–6 points)

• Environment/household safety: Interview-observed environmental or HH (inside/outside) risks (13 items)

The within-arm change in study outcomes from baseline to endline estimates the “intent to treat” effect of each intervention (Table 4). The magnitude of pre-post change in the RM and RMV-ECD intervention groups are estimated within arm by the proportion of caregivers who scored above the cutoff point (“good”) at endline. Those who scored poorly at both baseline and endline, or who decreased from good (baseline) to poor (endline) are categorized as “poor” at endline.

For the RM arm, only ECD knowledge (27.7% at baseline increasing to 33.6% at endline, *p* = .031) and environment/household risks (39.0% at baseline increasing to 57.1% at endline, *p* < .0001) were significantly improved after the intervention (RM: columns 2,5,6). In the RMV-ECD arm, all outcomes except environment/household risks significantly improved after the intervention with the largest change seen in ECD knowledge (increase of 35.8%, *p* < .0001) and the smallest change seen in father engagement (increase of 6.7%, *p* = .015; RMV-ECD: columns 3,7,8).

In Table 5, we show two adjusted logistic regression models on our study outcomes among caregivers from both study arms, examining the relative strength of association of the full RMV-ECD intervention compared to the RM intervention. The first model is adjusted for baseline level of the outcome and child age and the second model adds other variables of interest.

**Table 5:** RMV-ECD compared to RM only intervention: Adjusted logistic regression models

	Adjusted I – baseline status, child age			Adjusted II – add covariates		
	OR	(95% CI)	***p***-value	OR	(95% CI)	***p***-value
**Early child development knowledge**						
**RMV-ECD arm (ref: RM only)**	**4.90**	**(3.16, 7.59)**	**<.0001**	4.70	(3.07, 7.19)	<.0001
ECD knowledge score (0–6) at baseline	1.25	(1.17, 1.35)	<.0001	1.24	(1.15, 1.33)	<.0001
Child age in months	1.01	(0.99, 1.03)	0.390	1.01	(0.99, 1.03)	0.498
Assets (0–5)				**1.20**	**(1.01, 1.43)**	**0.039**
Parent Stress Index > 90 (endline)				**0.50**	**(0.35, 0.71)**	**0.0004**
**Early Stimulation practices**						
**RMV-ECD arm (ref: RM only)**	**1.88**	**(1.36, 2.58)**	**0.004**	2.09	(1.54, 2.83)	<.0001
ES practices score (0–1) at baseline	3.17	(1.54, 6.54)	0.003	2.72	(1.42, 5.19)	0.004
Child age in months	1.03	(1.00, 1.05)	0.031	1.02	(1.00, 1.05)	0.071
**Caregiver education**						
< Primary				1.00		
Primary complete				0.77	(0.50, 1.19)	0.234
Secondary+				**2.33**	**(1.57, 3.61)**	**0.0002**
Non-violent discipline score (endline)				**1.87**	**(1.58, 2.22)**	**<.0001**
**Father engage**^a^						
**RMV-ECD arm (ref: RM only)**	**1.90**	**(1.45, 2.48)**	**<.0001**	2.26	(1.71, 2.99)	<.0001
Father engage score (0–6) at baseline	1.42	(1.31, 1.54)	<.0001	1.43	(1.29, 1.58)	<.0001
Child age in months	0.99	(0.96, 1.01)	0.305	0.98	(0.95, 1.02)	0.307
Parent Stress Index > 90 (endline)				**0.72**	**(0.52, 0.99)**	**0.043**
Non-violent discipline score (endline)				**1.59**	**(1.34, 1.87)**	**<.0001**
**Responsive care**						
**RMV-ECD arm (ref: RM only)**	**1.21**	**(0.78, 1.86)**	**0.384**	1.05	(0.69, 1.59)	0.821
Responsive care scores (0–6) at baseline	1.05	(0.94, 1.15)	0.363	1.04	(0.93, 1.16)	0.478
Child age in months	0.96	(0.93, 0.99)	0.003	0.96	(0.93, 0.99)	0.015
Parent Stress Index > 90 (endline)				**0.31**	**(0.18, 0.54)**	**0.0001**
Violent discipline score (endline)				**0.88**	**(0.79, 0.97)**	**0.014**
**Environmental/household safety**						
**RMV-ECD arm (ref: RM only)**	**0.72**	**(0.51, 1.02)**	**0.064**	**0.77**	**(0.56, 1.43)**	**0.117**
Env/HH safety score (0–8) at baseline	1.01	(0.94, 1.08)	0.864	1.01	(0.93, 1.09)	0.881
Child age in months	1.00	(0.97, 1.02)	0.709	1.00	(0.97, 1.03)	0. 933
Lives in HH with extended family members				1.64	(1.32, 2.05)	<.0001
Injury requiring medical care, under-5 child in HH in past year				2.03	(1.51, 2.75)	<.0001
Child has had expected number of under-5 clinic visits since birth				0.77	(0.62, 0.96)	0.020

^a^Among caregivers with a partner

In model I, caregivers in the RMV-ECD arm were more likely than caregivers in the RM arm to score well in ECD knowledge (AOR 4.90, 95%CI: 3.16, 7.59), early stimulation (AOR 1.88, 95%CI: 1.36, 2.58), and father engagement (AOR 1.90, 95%CI: 1.45, 2.48), and a marginally significant protective association was found on environment risks. There was no observed association between the RMV-ECD arm and responsive care (AOR 1.21, 95%CI: 0.78, 1.86). In model II, we identified additional covariates that were significantly associated with the study outcomes. The common predictor across several study outcomes is parenting stress, where higher stress is associated with significantly lower likelihood of having good ECD knowledge (AOR 0.50, 95%CI: 0.35, 0.71), father engagement (AOR 0.72, 95%CI: 0.52, 0.99) and responsive care (AOR 0.31, 95%CI: 0.18, 0.54). Household assets (AOR 1.20, 95%CI: 1.01, 1.43) were associated with ECD knowledge and caregiver secondary education (AOR 2.33, 95%CI: 1.57, 3.61) was associated with early stimulation. Scoring higher (more likely) on the *non-violent* discipline score was associated with early stimulation (AOR 1.87, 95%CI: 1.58, 2.22) and father engagement (AOR 1.59, 95%CI: 1.34, 1.87); while a higher *violent* discipline score (psychological, physical and severe physical items) was associated with lower likelihood of responsive care (AOR 0.88, 95%CI: 0.79, 0.97). Factors associated with higher environment/household risks include living in an extended family household (AOR 1.64, 95%CI: 1.32, 2.05) and having a history of young child injury in the household (AOR 2.03, 95%CI: 1.51, 2.75). Caregivers whose children had the expected number of clinic visits (for their age) were less likely to score above the cutoff for environment/household risks (AOR 0.77, 95%CI: 0.62, 0.96).

## Discussion

In the full Malezi program intervention arm (RMV-ECD) with radio messaging, trained facility providers, and CHW-provided CCD-based counseling at home and facility sessions using video job aids, caregivers significantly improved in ECD knowledge, early stimulation, father engagement and responsive care, but there was no observed effect on reduced household environment risks. This suggests that a multilevel intervention has a greater association with study outcomes compared to a radio intervention with no additional health facility or community level engagement. Even after adjusting for baseline levels of the study outcomes and child age, caregivers in the full intervention arm were two to four times more likely to score well on ECD knowledge and early stimulation practice compared to caregivers in the radio-only arm. This finding is consistent with the recommendation that parenting interventions are most effective if they are integrated into existing health care systems coupled with provider training using an evidence-based curriculum and job aids. It is also important to provide opportunities for parents to practice behaviors and learn from provider feedback, all of which were components of the full Malezi program intervention [4, 12, 14]. Incorporating home-based ECD support, as the full Malezi program did, has also been key to some of the most successful ECD interventions [7, 20, 23]. However, a recent trial in India showed no difference between a home- and facility (group)-based ECD intervention, concluding that the group-based intervention was significantly more affordable than the home-based intervention [43].

The combined intervention had the strongest association with ECD knowledge, with equal but weaker relationships with early stimulation and father engagement, consistent with our conceptual model postulating that increased knowledge would partly, but not exclusively, drive parenting behavior change. One notable strength of this evaluation is the relatively high fidelity to the intervention as demonstrated by our data on high coverage of monthly CHW visits to caregiver’s homes, and frequent supervisory support (findings to be published separately). Several real-world evaluations of interventions requiring lay cadre to make home visits, particularly of programs implemented at scale [44], have faced challenges in ensuring coverage, intensity and quality of intervention delivery [45]. Another strength was the high follow-up rate of enrolled caregivers, with 94% follow-up, excluding those determined to have moved out of the area or died.

While we observed an improvement in responsive care in the combined intervention arm between baseline and endline, there was no change in the radio-only intervention on responsive care, nor was there an observed association with the combined intervention compared to the radio-only intervention. We cautiously interpret this finding to mean that there is weak or no evidence that either intervention improved responsive care behaviors. While some randomized trials have been able to improve responsive stimulation [21], infant attachment, or the mother-infant relationship [19], many other studies have only reported effects on parenting knowledge and practices [18, 22, 23, 46]. The concept of responsive care may be difficult for lay cadre to teach. CHWs may not have the confidence or skills to addresses responsive care, especially if they are more comfortable discussing the importance of play, communication, and specific ways parents could increase child stimulation. In a systematic review of parenting/ECD interventions, Jeong et al. describe several studies aiming to improve responsive care, and the methods used underscore the coaching intensity that may be required, with many employing recording of parent-child interactions to highlight and discuss opportunities for responsive caregiving [11].

Media communication campaigns can positively impact a wide range of child survival oriented parenting behaviors [25]. In Africa, given the widespread utilization of radio, these approaches are seen as effective tools for disseminating health information and supporting health-promoting behaviors [27], although effects of media-based interventions on behavioral endpoints are generally small [47, 48]. In this study, the radio intervention alone was not linked to observed shifts in parenting behaviors, but it was linked to improved ECD knowledge among caregivers and household safety. This is an encouraging finding and suggests that investing in mass media to promote ECD messaging could be an affordable and broad-based intervention with small but potentially important effects on the population, ideally reinforced through community-based health communications. Conversely, no reduction in household environment risks was observed in the combined intervention group. This may be explained by the higher proportion of caregivers in the radio-only group having such risks at baseline compared to caregivers in the combined intervention group. It is possible that the effect of the Malezi I program had already reduced risks among caregivers in the combined intervention group to a point where further reductions could be hard to achieve, while caregivers in the radio-only group were able to “catch up” after being exposed to radio messaging about ECD and child safety measures.

The addition of a multi-media component to a comprehensive health system-based intervention makes this intervention unique and has likely contributed meaningfully to the overall changes observed in the full intervention arm. The relatively small changes in the radio-only arm, compared with the larger changes seen in the full intervention arm suggest that media messages heard by caregivers need reiteration, explanation and practical coaching to be sufficiently reinforced to drive parental behavior change [12]. The ECD video job aids, which illustrated messages in a video shown by tablet, allowed the CHW to stop, highlight and replay sections of the video for discussion. The videos also helped with delivery of standardized and complete ECD messages, adherent to the CCD curriculum.

This study highlights the importance of two psychosocial-behavioral factors -- parenting stress and discipline practices. Caregivers scoring above the cutoff for parenting stress were significantly less likely to score well on ECD knowledge, father engagement and responsive care. A study from Ghana reported that stress was positively associated with depression among mothers with comparable levels of parenting stress as in this study [41]. Parenting stress may be conceptualized as a state whereby the caregiver does not have the emotional capacity or skills to cope with the cumulative demands of parenting leading to physical and emotional fatigue. Thus, parenting stress may deplete mental energy required to be emotionally supportive and provide developmental stimulation to the child. Studies show that higher parenting stress is related to less nurturing behavior, decreased enjoyment and increased conflict/punitive practices [49]. However, higher levels of perceived service availability have been shown to mediate the relationship between parenting stress and child neglect [50].

Non-violent discipline practices were associated with increased likelihood of early stimulation and father engagement, while violent discipline practices were associated with lower likelihood of responsive care. Discipline practices are a core parenting behavior and likely to be directly related to early stimulation and responsive care practices [51], although cultural differences in views of parental authority and respect for elders also shape discipline practices [52]. Embracing positive instead of violent disciplinary practices is an important component of nurturing care: ensuring the child feels safe from emotional or physical abuse [53].

The Malezi program intervention did not include substantive messages or focused training of CHWs on how to support caregivers to positively discipline their child or cope with parenting stress, a notable content gap in many parenting interventions [11]. However, training providers to identify signs of high parenting stress or violent discipline practices, and to mentor caregivers more directly in positive discipline and managing stress, could have measurable effects on parenting behaviors and child development outcomes. One study found that peer-led group caregiver support intervention (12 fortnightly sessions) led to reduced depressive symptoms in mothers [8], and depression is likely correlated with parenting stress. UNICEF is also now promoting caregiver mental health as a training module to accompany CCD interventions, called Caring for the Caregiver (https://www.unicef.org/documents/caring-caregiver).

One limitation to this study was the reliance on self-reported measures for early stimulation and (mostly maternal) primary caregiver-reported father engagement. Similarly, while our responsive care measure was derived from observations during the interview, a more robust approach to data collection would have been to observe a standardized caregiver-child interaction session. Another limitation is that while we measured discipline practices and parenting stress at baseline and endline, we chose to associate the endline measures with our study outcomes because they were more proximate to the caregiver’s recent behavior or state of mind. However, this means the observed associations are not established as causal factors, and it is possible that parenting stress may be caused by lower ECD knowledge or limited practice of child stimulation behaviors. A third limitation is that this study focused outcome measures on the primary caregiver, even though it is widely understood that father’s engagement and extended family influencers are important factors in child-rearing norms and practices within households and the community. While the radio-message component of our interventions was broad-based, designed to target all potential caregivers, the focus of the CHWs was mostly on the primary caregiver, who was almost always the biological mother. Finally, this study was designed as a program evaluation with a relatively short intervention period. Thus, we intentionally focused on proximate outcomes -- parenting knowledge and behaviors -- rather than child development outcomes.

## Conclusions

This study represents a rigorous real-world program evaluation of an ECD project in Tanzania. An intervention that includes mass media, educational video content and CHWs who counsel and mentor caregivers in their homes and at health facilities was associated with significant improvements in ECD parenting knowledge and behaviors but a relationship with responsive care could not be established. A broader-based mass media-only approach, using radio messaging, will likely support improved ECD knowledge and reduced environment/household risks. The role of parenting stress and caregiver approaches to discipline were identified as key factors associated with ECD knowledge, early stimulation, father engagement and responsive care. Community-based parenting interventions targeting even the youngest children of 0–3 years could be improved by incorporating more messaging and caregiver coaching in positive discipline and managing parental stress. Future program design could also benefit from research that inquires more deeply into fathers’ roles in creating a nurturing and safe environment for children.

## Data Availability

The datasets used and/or analyzed during the current study are available from the corresponding author on reasonable request.

## Abbreviations

AOR: Adjusted odds ratio
CCD: Care for Child Development (UNICEF)
CHW: Community health worker
CI: Confidence interval
ECD: Early child development
IQR: Inter-quartile range
MICS: Multiple Indicator Cluster Surveys (UNICEF)
OR: Odds ratio
PSI: Parenting Stress Index (scale)
RM: Radio messaging
RMV-ECD: Radio messaging, video, early child development
SD: Standard deviation
